# Diagnosis and treatment modalities of hilar biliary duct stricture in hepatic cystic echinococcosis after endocystectomy

**DOI:** 10.1051/parasite/2021051

**Published:** 2021-06-18

**Authors:** Paizula Shalayiadang, Abduaini Abulizi, Ayifuhan Ahan, Tiemin Jiang, Bo Ran, Ruiqing Zhang, Qiang Guo, Hao Wen, Yingmei Shao, Tuerganaili Aji

**Affiliations:** 1 Department of Hepatobiliary and Echinococcosis Surgery, Digestive and Vascular Surgery Center, First Affiliated Hospital of Xinjiang Medical University 830054 Urumqi Xinjiang PR China; 2 State Key Laboratory of Pathogenesis, Prevention and Management of High Incidence Diseases in Central Asia, First Affiliated Hospital of Xinjiang Medical University 830054 Urumqi Xinjiang PR China

**Keywords:** Hepatic cystic echinococcosis, Endocystectomy, Hilar biliary duct stricture, Diagnosis, Treatment

## Abstract

*Aim*: Hilar biliary duct stricture may occur in hepatic cystic echinococcosis (CE) patients after endocystectomy. This study aimed to explore diagnosis and treatment modalities. *Methods*: Clinical data of 26 hepatic CE patients undergoing endocystectomy who developed postoperative hilar biliary duct stricture were retrospectively analyzed and were classified into three types: type A, type B, and type C. Postoperative complications and survival time were successfully followed up. *Results*: Imaging showed biliary duct stenosis, atrophy of ipsilateral hepatic lobe, reactive hyperplasia, hepatic hilum calcification, and dilation or discontinuity of intrahepatic biliary duct. All patients received partial hepatectomy to resect residual cyst cavity and atrophic liver tissue, and anastomosis of hepatic duct with jejunum or common bile duct exploration was applied to handle hilar biliary duct stricture. Twenty-five patients were successfully followed up. Among type A patients, one patient died of organ failure, and upper gastrointestinal bleeding and liver abscess occurred in one patient. Moreover, calculus of intrahepatic duct was found in one type B and type C patient. *Conclusion*: Long-term biliary fistula, infection of residual cavity or obstructive jaundice in hepatic CE patients after endocystectomy are possible indicators of hilar bile duct stricture. Individualized and comprehensive treatment measures, especially effective treatment of residual cavity and biliary fistula, are optimal to avoid serious hilar bile duct stricture.

## Abstract (fr)

*Objectif* : une sténose du canal biliaire hilaire peut survenir chez les patients atteints d’échinococcose kystique (EK) hépatique après une endocystectomie. Cette étude visait à explorer les modalités de diagnostic et de traitement. *Méthodes* : Les données cliniques de 26 patients atteints de EK hépatique subissant une endocystectomie et ayant subi une sténose du canal biliaire hilaire postopératoire ont été analysées rétrospectivement, et ont été classées en trois types (type A, type B, type C). Les complications postopératoires et le temps de survie ont été suivis avec succès. *Résultats* : L’imagerie a montré une sténose du canal biliaire, une atrophie du lobe hépatique homolatéral, une hyperplasie réactive, une calcification du hile hépatique, une dilatation ou une discontinuité du canal biliaire intrahépatique. Tous les patients ont subi une hépatectomie partielle pour réséquer la cavité kystique résiduelle et le tissu hépatique atrophique, et une anastomose du canal hépatique avec le jéjunum ou une exploration du canal cholédoque a été appliquée pour traiter la sténose du canal biliaire hilaire. Vingt-cinq patients ont été suivis avec succès. Parmi les patients de type A, un patient est décédé d’une insuffisance organique et un saignement gastro-intestinal supérieur et un abcès hépatique sont survenus chez un patient. De plus, un calcul du canal intrahépatique a été trouvé chez un patient de type B et de type C. *Conclusion* : Une fistule biliaire à long terme, une infection de la cavité résiduelle ou une jaunisse obstructive chez les patients atteints d’EK hépatique après endocystectomie sont des indicateurs possibles de sténose du canal biliaire hilaire. Des mesures de traitement individualisées et complètes, en particulier un traitement efficace de la cavité résiduelle et de la fistule biliaire, sont optimales pour éviter une sténose grave des voies biliaires hilaires.

## Introduction

Cystic echinococcosis (CE), a widely distributed zoonosis, still continues to be a significant economic burden and public health issue, causing an average of 285,500 disability-adjusted life years [7–9]. CE cysts mainly develop in the internal organs of humans or other intermediate hosts, with the liver and lungs being the most common target organs [12, 30]. Based on cyst type, classification, size, location, presence of complications and available medical expertise and equipment, various options are possible for the treatment of hepatic CE, including anti-infective drugs, surgery, non-surgical interventions, and a watch-and-wait approach [6]. To prevent relapse, periadventitial cystectomy (total cystectomy) is a preferred choice in clinical settings. If CE cysts are adjacent to major vessels, sub-total cystectomy is also considered without vessel dissection. When the CE lesion is huge and close to vessels or biliary ducts of porta hepatis, endocystectomy (partial cystectomy) may be feasible [16, 28].

In the long-term expansive growing process, CE cysts compress liver tissue, the surrounding intrahepatic vessels and bile ducts, and biliary communication in the first porta hepatis may form a fistula of the cystic cavity and bile ducts, which is the main cause of postoperative recurrence and secondary infection [29]. Due to residual cyst cavity and postoperative biliary leakage after endocystectomy, hepatic CE patients may develop postoperative complications of fistula, remnant cavity infection, and even severe hilar biliary stricture, which is a major challenge for surgeons in clinical practice [1, 26]. Therefore, accurate diagnosis and timely treatment of postoperative complications, especially hilar biliary stricture, are essential for hepatic CE patients who underwent endocystectomy. This study aimed to summarize clinical features, and diagnostic and treatment modalities of 26 hepatic CE cases developing hilar biliary stricture after endocystectomy, which may provide valuable references for medical professionals in their clinical work.

## Materials and methods

### Ethics

Design of this study and management of patients and controls were in accordance with the Helsinki Declaration and approved by the Human Ethics Committee of the First Affiliated Hospital of Xinjiang Medical University [32]. Written and signed informed consent was obtained from all patients or their legal representatives in their native language. Patients under the age of 18 were not included in this study.

### Patients

We conducted a retrospective data analysis of 26 patients diagnosed with hepatic CE who underwent endocystectomy at the First Affiliated Hospital of Xinjiang Medical University from January 2005 to January 2018. In addition, the patients were accompanied by hilar biliary duct stricture after endocystectomy. The patients’ medical records were analyzed for demographic data, previous surgical history, clinical symptoms, therapeutic method, postoperative complications, and other follow-up data.

### Assessment and classification of hilar biliary duct stricture

**Figure 1:**
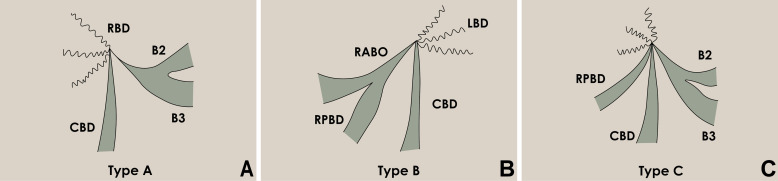
Diagram for different types of hilar biliary duct structure. (A) Diagram for type A hilar biliary duct structure. (B) Diagram for type B hilar biliary duct structure. (C) Diagram for type C hilar biliary duct structure.

**Figure 2:**
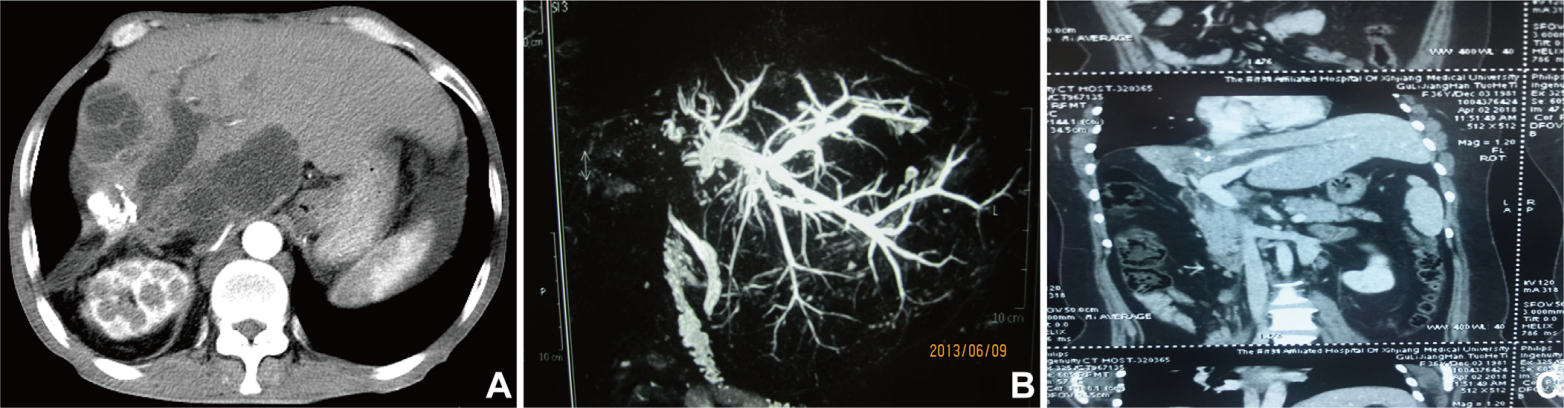
Representative imaging for type A hilar biliary duct stricture. (A) Abdominal enhanced CT demonstrating dilation of left hepatic duct caused by hilar calcification, hyperplasia of left hepatic lobe and two recurrent CE lesion. (B) Abdominal MRI indicating obvious dilation of left hepatic duct and discontinuity of portal hepatis. (C) Abdominal CT showing atrophy of right lobe and dilation of left hepatic duct.

**Figure 3:**
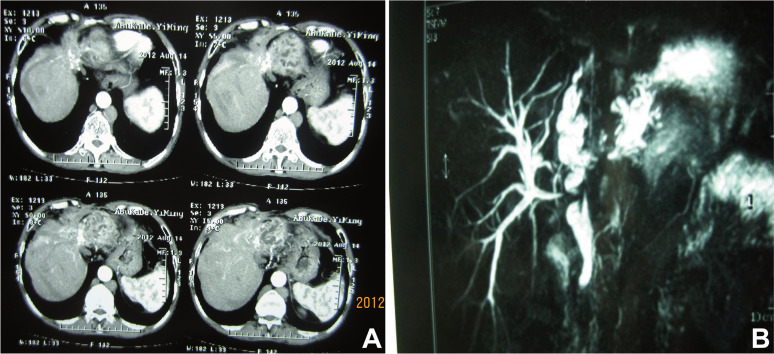
Representative imaging for type B hilar biliary duct stricture. (A) Abdominal enhanced CT demonstrating atrophy of left hepatic lobe caused by calcification of outer laminated layer, hyperplasia of right hepatic duct. (B) Abdominal MRI showing severe hilar biliary duct stricture, obvious dilatation of left hepatic duct and traction stricture of the right bile duct.

**Figure 4:**
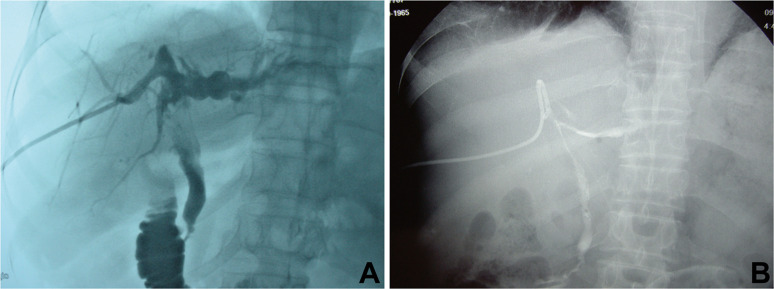
Representative imaging for type C hilar biliary duct stricture. (A) Preoperative cholangiography showing upward retraction of the right anterior hepatic duct orifice caused by CE residual cavity and transverse of the left hepatic duct. (B) Postoperative cholangiography indicating that the angle between right hepatic duct and common hepatic duct was still large.

Abdominal ultrasound, computed tomography (CT), magnetic resonance imaging (MRI), and transhepatic cholangiography were used for assessment and classification of hilar biliary duct stricture after surgery. Preoperative percutaneous transhepatic biliary drainage (PTBD) was performed in patients whose total bilirubin level was above 129.1 μmol/L. Based on the Bismuth–Corlette classification of hilar biliary duct stricture [23], the patients were classified into three types according to the representative imaging and intraoperative findings. Residual cavity of hydatid and atrophy occurred in the right hepatic lobe, and right hepatic duct stenosis caused traction stricture of the left bile duct and common hepatic duct. Moreover, hilar biliary duct was dragged to the right hepatic lobe, which was categorized into type A and was analogue to type IIIa Bismuth–Corlette classification (Figs. 1A and 2). In comparison, residual cavity and atrophy was found in the left hepatic lobe, and left hepatic duct stenosis resulted in traction stricture of the right bile duct and common hepatic duct. Additionally, hilar biliary duct was pulled to the left hepatic lobe, which was classified into type B and was analogue to type IIIb Bismuth–Corlette classification (Figs. 1B and 3). Moreover, residual cavity and atrophy were located in the middle hepatic lobe (mainly in the fourth, fifth and eighth hepatic segment), and upward traction stricture of the right anterior segmental duct or combined with stricture of common hepatic duct, left hepatic duct and right hepatic duct occurred. In addition, hilar biliary duct was dragged to the right anterior segment, which classified into type C and was analogue to type IV Bismuth–Corlette classification (Figs. 1C and 4).

### Postoperative follow-up

Long-term follow-up was conducted for the patients due to relative high relapse rates and postoperative complications of CE, as recommended [17]. The abdominal ultrasound/CT and liver functions were reviewed every three months within one year, and every six months after one year in order to evaluate postoperative complications. Postoperative complications were evaluated based on the Clavien–Dindo classification system [11]. Patients’ follow-up data were collected through outpatient review and/or telephone. The patients were followed up from January 2005 and the deadline was January 2018 or death time of the patients.

### Statistical analysis

Statistical analysis was performed using the Statistical Package for Social Science (SPSS) version 21.0 (SPSS Inc., Chicago, IL, USA). All quantitative data were presented as the mean ($\bar{X}$) ± standard deviation (SD).

## Results

### Characteristics of the patients

All hepatic CE patients included in this study underwent endocystectomy from January 2005 to January 2018, and there were postoperative complications of hilar biliary duct stricture. In this study, all the patients underwent endocystectomy for hepatic CE lesions in the local hospitals for the first time. When they developed postoperative complications of hilar biliary duct stricture, the patients then further sought treatment in our department. Thus, it was not appropriate to precisely indicate the patient numbers who underwent endocystectomy and the rate of biliary stricture after surgery. There were 17 male and nine female patients, whose ages ranged from 25 to 70 years, with the median age of 45.42 ± 11.20 years. All the participants had previous operation surgery for hepatic CE. Among them, 14 patients (53.85%) had one operation, eight patients (30.77%) had two operations, two patients (7.69%) had three operations, and two patients (7.69%) had four operations. All patients with one operation received endocystectomy. Patients with two or more operations underwent endocystectomy at the first operation time, and common bile duct exploration and lithotomy were selected as treatment options at later times. Among the patients, 18 underwent endocystectomy at the first time in local hospitals due to poor technical conditions, and residual cavity was not treated properly. Thus, the 18 cases (69.2%) had long-term biliary fistula. The longest time for abdominal drainage tube usage due to biliary fistula was one year. Nine patients (34.61%) developed residual cavity infection; thus, the drainage tubes were replaced several times. Moreover, endoscopic retrograde cholangiopancreatography (ERCP) was applied for biliary stent implantation in four patients (15.39%). Additionally, biliary fistula was spontaneously closed in five patients (19.23%) within three months.

### Clinical manifestations

Among the 26 patients, six patients (23.08%) had main symptoms of abdominal pain and jaundice, two patients (7.69%) had jaundice and fever, three patients (11.54%) had abdominal pain and fever, 12 patients (46.15%) had obstructive jaundice, two type B patients (7.69%) had long-term non-union of skin sinuses, and one type C patient (3.85%) had bile expectoration. Detailed clinical manifestations of the patients are shown in Table 1.

**Table 1:** Clinical manifestations and detailed surgical aspects.

Characteristics	Type A (*n* = 10)	Type B (*n* = 10)	Type C (*n* = 6)
Clinical manifestations			
Abdominal pain and jaundice	2	3	1
Jaundice and fever	1	0	1
Abdominal pain and fever	1	1	1
Jaundice	6	4	2
Skin sinuses	0	2	0
Bile expectoration	0	0	1
Treatment modalities			
Hepatectomy and anastomosis of hepatic duct with the jejunum	2	5	0
Hepatectomy and artificial stent implantation of hepatic duct	2	5	3
Common bile duct exploration and artificial stent implantation of hepatic duct	6	0	0
Hepatectomy and common bile duct exploration	0	0	2
Hepatectomy combined with superior cholangiojejunostomy	0	0	1
Operation time (h)	3.95 ± 1.40	4.55 ± 0.96	5.92 ± 2.97
Blood loss (mL)	617.00 ± 601.39	480.00 ± 396.65	900.00 ± 976.37
Blood transfusion (U)	2.30 ± 3.95	1.40 ± 2.12	3.17 ± 1.94

### Diagnostic methods

Routine laboratory examinations including liver function, routine blood test, and coagulation profiles demonstrated jaundice and different degrees of liver function damage in most patients. Abdominal CT scan showed that there occurred biliary duct stenosis and atrophy of the ipsilateral hepatic lobe. In addition, there may also be reactive hyperplasia and calcification of the hepatic hilum (Figs. 2A, 2C and 3A). MRI demonstrated that the intrahepatic biliary duct was dilated, and that there may be hilar biliary duct defect or discontinuity of intrahepatic biliary ducts. Moreover, severe hilar biliary duct deformation may also be found in some patients. The distance between the common hepatic duct and dilated intrahepatic biliary duct above the stenosis became longer and the angle became larger (Figs. 2B and 3B). In addition, cholangiography showed that the hepatic duct was retracted or transversed due to long-term compression by the CE residual cavity, and transverse of the left hepatic duct. The angle between intrahepatic ducts and the common hepatic duct may become larger (Figs. 4A and 4B).

### Treatment modalities

**Figure 5:**
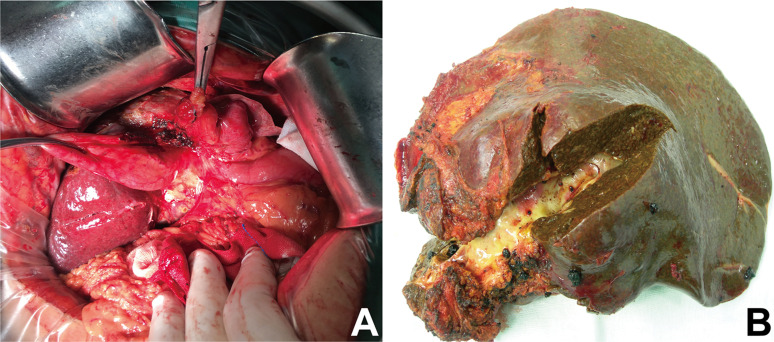
Typical intraoperative photos. (A) Intraoperative exposition of hilar biliary duct stricture. (B) Specimens showing obvious hilar biliary duct stricture and scar formation.

Detailed surgical aspects for the patients are shown in Table 1. Among the patients, seven underwent hepatectomy and anastomosis of the hepatic duct with the jejunum. Ten underwent hepatectomy and artificial stent implantation of the hepatic duct. In addition, common bile duct exploration and artificial stent implantation of the left hepatic duct was performed in six type A patients. Two type C patients underwent right anterior lobe hepatectomy and common bile duct exploration, and one type C patient underwent middle lobe hepatectomy combined with superior cholangiojejunostomy. Average surgical time for type A patients was 3.95 ± 1.40 h, and average intraoperative blood loss was 617.00 ± 601.39 mL. Four patients needed blood transfusion and average blood transfusion was 5.75 ± 4.50 U. Average surgical time for type B patients was 4.55 ± 0.96 h, and average intraoperative blood loss was 480.00 ± 396.65 mL. Four patients needed blood transfusion and average blood transfusion was 3.50 ± 1.92 U. Average surgical time for type C patients was 5.92 ± 2.97 h, and average intraoperative blood loss was 900.00 ± 876.36 mL. All the patients needed blood transfusion and average blood transfusion was 3.17 ± 1.94 U. Typical intraoperative photos are shown in Figure 5. Biliary fistula was still found in nine patients, including two type A, four type B, and three type C patients after surgery.

### Follow-up data

Among the patients involved in the study, 25 patients were successfully followed up (96.15%). One type C patient was lost to follow up and the missed rate was 3.85%. Average follow-up time for type A, type B and type C patients was 69.70 ± 45.62 months, 66.10 ± 24.81 months, and 104.17 ± 17.05 months, respectively. During follow-up, one type A patient died of organ failure two years after surgery, and upper gastrointestinal bleeding was founded in one patient after one year, which was treated with conservative therapies. Additionally, repeated liver abscess occurred in one patient. Moreover, calculus of the intrahepatic duct was found in one type B and one type C patient. Detailed follow-up data are shown in Table 2.

**Table 2:** Detailed follow-up data.

Follow-up details	Type A (*n* = 10)	Type B (*n* = 10)	Type C (*n* = 6)
Patient number	10	10	5
Follow-up time (month)	69.70 ± 45.62	66.10 ± 24.81	104.17 ± 17.05
Clavien–Dindo classification			
<IIIa	1	1	1
≥IIIa	1	0	0

## Discussion

In recent years, surgical procedures of hepatic CE have been improved significantly. In our center, based on cyst type, location, size and presence/absence of complications, treatment modalities of total cystectomy, subtotal cystectomy, hepatectomy and endocystectomy were used for hepatic CE patients [31]. Based on our experience and other studies [14, 24], hepatectomy may be used as a preferred treatment modality in the following situations: (a) several CE cysts were confined to one hepatic segment or hepatic lobe; (b) there were recurrent thick wall cysts accompanied by intracystic infection or granuloma formation; and (c) there was biliary fistula in the remnant cavity with long-term tube or repeated debridement without recover. When hepatic CE lesions are localized in special sites and their volume is huge, it may sometimes be risky to perform total cystectomy. Therefore, endocystectomy is still the preferred surgical procedure in clinical settings [13, 21]. Western China is one of the endemic areas of echinococcosis, and about 100 hepatic patients may receive surgery for the removal of CE lesions annually, among which half of the patients underwent endocystectomy. Nevertheless, postoperative complications of repeated biliary fistula, obstructive jaundice, residual cavity infection, and even hilar biliary duct stenosis may occur subsequently. Due to its complete difference from common hilar biliary duct stenosis caused by cholangitis, treatment of hilar biliary duct stenosis induced by hepatic CE after surgery is extremely difficult and is a major challenge for medical staff.

With expansive growth of CE cysts, surrounding liver tissue, intrahepatic vessels and bile ducts are compressed, and a biliary duct fistula may be formed due to necrosis of bile duct walls [5, 10]. Fistula between the CE cyst and larger intrahepatic bile ducts may occur due to rupture of CE cysts located in the middle hepatic lobe or near the first hepatic hilum (mainly in the first, third, fourth and fifth hepatic segment) [4, 20]. Most CE cysts were located near major vessels and biliary ducts. As a result, total cystectomy has higher risks and endocystectomy is relatively feasible under these circumstances. However, this operation always remains part or all laminated layers that communicate with the hilar biliary duct, resulting in refractory complications of repeated residual cavity fistula and infection, whose incidence may range from 10.8% to 65.8% [2]. If biliary fistula is not treated properly during endocystectomy, it may cause serious bile leakage and then residence time of the drainage tube may be long, which conversely results in retrograde infection of the residual cavity. Then, patients need multiple puncture drainage or surgical treatment. During the repeated infection and healing process of residual cavity, fibrous tissue of the laminated layer shrinks continuously, and hilar biliary duct connected with the laminated layer twists, thickens and eventually forms stricture. Meanwhile, bile reflux on the stenosis side is also affected, which may then cause atrophy of liver tissue. In severe circumstances, hilar biliary duct stricture of the contralateral hepatic lobe and stones are formed [18]. In this study, we summarized the following characteristics of hilar biliary duct stricture in hepatic CE patients undergoing endocystectomy: (a) biliary fistula and repeated residual cavity infection were the main causes of hilar biliary duct stricture, whose healing time was long; (b) calcification of the cyst wall in the residual cavity was obvious, which was not conducive to biliary fistula healing; (c) in some cases, biliary duct stricture coexisted with intrahepatic biliary duct stones, which caused infection and stricture; and (d) in some cases, scar tissue around the hilar biliary duct directly compressed the portal vein or hepatic hyperplastic atrophic syndrome caused portal vein distortion, and even hilar biliary duct stricture accompanied by portal hypertension.

Diagnosis of hilar biliary stricture induced by hepatic CE surgery may include the following aspects: (a) first symptoms of most patients were similar with symptoms of cholangitis, including obstructive jaundice, abdominal pain and fever. In some circumstances, manifestations of hematemesis, long-term non-union of skin sinuses, and bile expectoration may also be seen [33, 34]; (b) abdominal CT scan may show biliary duct stenosis and atrophy of the ipsilateral hepatic lobe. In addition, there may also be different degrees of biliary duct stenosis, reactive hyperplasia of hepatic lobe, and calcification of the hepatic hilum; and (c) MRI may demonstrate dilatation of the intrahepatic biliary duct, severe hilar biliary duct deformation, defect of the hilar biliary duct, or discontinuity of intrahepatic biliary ducts. The distance between the common hepatic duct and dilated intrahepatic biliary duct above the stenosis becomes longer and the angle becomes larger [3]. Due to its rarity, most cases were misdiagnosed or treated improperly in unspecialized hospitals; therefore, most causes of hilar biliary duct stricture were not explored [25]. In this study, ten patients with co-existing hilar biliary duct stricture and intrahepatic biliary duct stones underwent common bile duct exploration and T-tube drainage several times. Nine patients with residual cavity infection underwent repeated puncture drainage or replacement of drainage tube, and two patients with skin sinus also had repeated sinus resection without considering possible causes. Combined with the Bismuth–Corlette classification of biliary duct stricture and according to representative imaging and intraoperative findings, hilar biliary duct strictures of the patients were classified into three types in this study. Bismuth–Corlette classification was beneficial to precisely locate the biliary duct stricture [23]. Classification of biliary duct stricture applied in this study greatly improved based on the type III and type IV Bismuth–Corlette classification, and the significant difference was traction of hilar biliary duct towards the residual cavity of hydatid. To be specific, residual cavity of hydatid and atrophy occurred in the right hepatic lobe, and right hepatic duct stenosis caused traction stricture of the left bile duct and common hepatic duct. Moreover, hilar biliary duct was dragged to the right hepatic lobe, which was classified into type A. In comparison, residual cavity and atrophy was located in the left hepatic lobe, and left hepatic duct stenosis resulted in traction stricture of the right bile duct and common hepatic duct. Additionally, hilar biliary duct was pulled to the left hepatic lobe, which was classified into type B. Moreover, residual cavity and atrophy was found in the middle hepatic lobe (mainly in the fourth, fifth and eighth hepatic segment), and there was upward traction stricture of the right anterior segmental duct or combined with stricture of common hepatic duct, left hepatic duct and right hepatic duct. Then, hilar biliary duct was dragged to the right anterior segment, which was classified in type C.

Curative treatment of hilar biliary duct stricture after hepatic CE surgery is achieved through completely removing the atrophic hepatic lobe and maintaining patency of the remaining biliary ducts. Clinically, it is found that contraction of the residual cavity after hepatic CE surgery has effects on scar formation of the hilar biliary duct, which is always covered by hypertrophic liver tissue. Additionally, hepatic rotation may cause some changes of hilar duct position and cause problems for resection of atrophic liver tissue and exposition of dilated bile duct above the contralateral stricture [22, 33]. Based on our experience on the treatment of 26 patients, it is suggested that individualized treatment should be considered according to the patients’ clinical condition. (a) For patients with severe ipsilateral biliary duct stricture combined with liver tissue atrophy, if the distance between the residual cavity and first porta hepatis is longer and contralateral intrahepatic duct stenosis is relatively mild, resection of the atrophic hepatic lobe combined with contralateral cholangiojejunostomy is the ideal surgical method. Compared with type A patients, most type B patients can perform this surgical procedure (left hepatic lobe resection and right hepatic duct jejunostomy). (b) For patients with severe ipsilateral biliary duct stricture combined with significant atrophy of liver tissue, when the distance between the residual cavity and first porta hepatis is near and the contralateral intrahepatic duct stenosis is relatively severe, contralateral cholangiojejunostomy is impossible due to difficulty in exposition of the normal hepatic biliary duct. Thus, resection of the atrophic hepatic lobe and artificial stent implantation of the contralateral biliary duct is feasible. Compared with type A and C patients, more type B patients received this surgical procedure. After resection of left atrophic liver tissue, the right hepatic duct was pulled and twisted in type B patients. However, right liver position was relatively shallow and only changed between the first and second hepatic hilus, whose position was still shallow. Thus, removing scar tissue around the biliary duct, correcting the twisted angle, and inwardly inserting an artificial stent into the duodenum to form biliary stent drainage may effectively solve the obstruction. (c) For patients with severe ipsilateral biliary duct stricture combined with significant atrophy of liver tissue, when the residual cavity was located above the first porta hepatis, hyperplasia and hypertrophy occurred in contralateral liver tissue and contralateral intrahepatic duct stenosis is relatively severe, identifying common hepatic duct and artificial stent implantation of contralateral biliary duct is reasonable. In this study, most type A patients underwent this surgical procedure. Residual cavity was directly connected with the right hepatic duct. After surgery, the right hepatic lobe atrophied, but the left lobe became hypertrophic, which results in rotation of liver to the right and then covering the porta hepatis. Meanwhile, rotation of the hepatoduodenal ligament deepened biliary duct position. The portal vein was also pulled in some patients, which caused portal hypertension and establishment of collateral circulation around biliary duct. During the operation, the common hepatic duct was successfully identified in four patients, and then an artificial stent was inserted into the left hepatic duct. Two patients underwent PTBD before the operation, and artificial sent was successfully inserted into the stricture segment through using D tube.

Treatment procedures of hilar biliary duct stricture after hepatic CE surgery is a difficult and complicated task, and there may also be massive complications after surgery. Among the patients, nine had complications during perioperative period or hospitalization, and five had complications during postoperative follow-up, including one who died of hepatic failure. Therefore, surgeons should give more attention to the prevention of hilar biliary duct stricture formation in clinical practice. It is important to deal with residual biliary fistula in a proper manner when hepatic CE patients are complicated with biliary duct rupture [15, 19]. If the facilities and expertise are available, total cystectomy combined with suture of biliary fistula is strongly recommended. Moreover, it is also preferred to perform endocystectomy combined with suture of biliary fistula after inserting appropriate T-tube into the residual cavity and place another T-shaped drainage tube in the common biliary duct [27]. Resection of hepatic lobe not only avoid formation of biliary fistula and repeated infection of residual cavity, but it may also effectively prevent hilar biliary duct stricture. This study had several limitations. Due to the retrospective nature, there may be some bias. Moreover, the high number of losses to follow-up may also affect information on long-term outcomes. Importantly, due to poor technical conditions of local hospitals and late arrival to more specialized hospitals, the initial surgical technique selected in the patients was not appropriate. In this regard, large clinical studies are needed to provide more accurate diagnostic and treatment modalities for hepatic CE patients with postoperative complications.

## Conclusion

To sum up, postoperative hepatic CE patients with long-term biliary fistula, residual cavity infection, or obstructive jaundice should consider the possibility of hilar biliary duct stenosis. Since this clinical picture is more complex and involves high risk, surgeons should carry out individualized and comprehensive treatment measures according to patients’ clinical characteristics. Importantly, it is crucial to properly deal with biliary fistula in the residual cavity when performing endocystectomy at the first time, which is of great significance in avoiding serious complications of hilar biliary duct stricture.

## Conflict of interests

The authors declare that they have no conflict of interest.

## Author contributions

Paizula Shalayiadang: Conception and design; acquisition of data; analysis and interpretation of data; drafting the article.

Abduaini Abulizi: Analysis and interpretation of data; drafting the article.

Ayifuhan Ahan: Acquisition of data; analysis and interpretation of data.

Tiemin Jiang: Acquisition of data; analysis and interpretation of data.

Bo Ran: Acquisition of data; analysis and interpretation of data.

Ruiqing Zhang: Acquisition of data; analysis and interpretation of data.

Qiang Guo: Acquisition of data; analysis and interpretation of data.

Hao Wen: Conception and design; provision of study material.

Yingmei Shao: Conception and design; provision of study material.

Tuerganaili Aji: Conception and design; final approval of the version to be submitted.
